# The Closed-Can Exhalation Method for Measuring Radon

**DOI:** 10.6028/jres.095.019

**Published:** 1990

**Authors:** Christer Samuelsson

**Affiliations:** Department of Radiation Physics, University of Lund, University Hospital, S 221 85 Lund, Sweden

**Keywords:** closed-can method, exhalation, radon, radon diffusion

## Abstract

Results from closed-can radon exhalation experiments must be interpreted bearing the time-dependent radon diffusion theory in mind. A rapid change from the free to final steady-state exhalation rate will take place for all samples that are thin compared with the radon diffusion length. The radon gas accumulating in a closed can corresponds to a free exhalation rate only if the outer volume of air is much larger than the pore volume of the enclosed sample, or the thickness of the sample is much larger than the radon diffusion length.

## 1. Background

The closed-can method is a well-known and established way of measuring radon outgassing from porous samples. Unfortunately, the interpretation of experimental results has frequently been based on assumptions that are in conflict with the basic laws of diffusion. The erroneous and, one could say, classical approach is to fit the radon build-up in the accumulator to the equation

$$A=\frac{E}{\lambda }(1-e^{-\lambda t})$$ (1)

where

*A* = radon activity in enclosure (Bq),
λ = decay constant of radon-222 (s^−1^),
*E* = exhalation rate (Bq s^−1^),
*t* = duration of enclosure (s).

It is assumed without evidence that the exhalation *E* is initially constant, typically for several hours after closing the accumulator. Diffusion theory calculations show that this is not true for samples which are thin compared with the diffusion length. Including effects of leakage and the so-called “back diffusion” in eq (1) by incorporating an “effective” decay constant λ^*^ instead of λ is even more suspect. Equation (1) is typical for first-order kinetics and cannot successfully be imposed on pure diffusive transportation processes.

## 2. Results and Discussion

**Figure 1:**
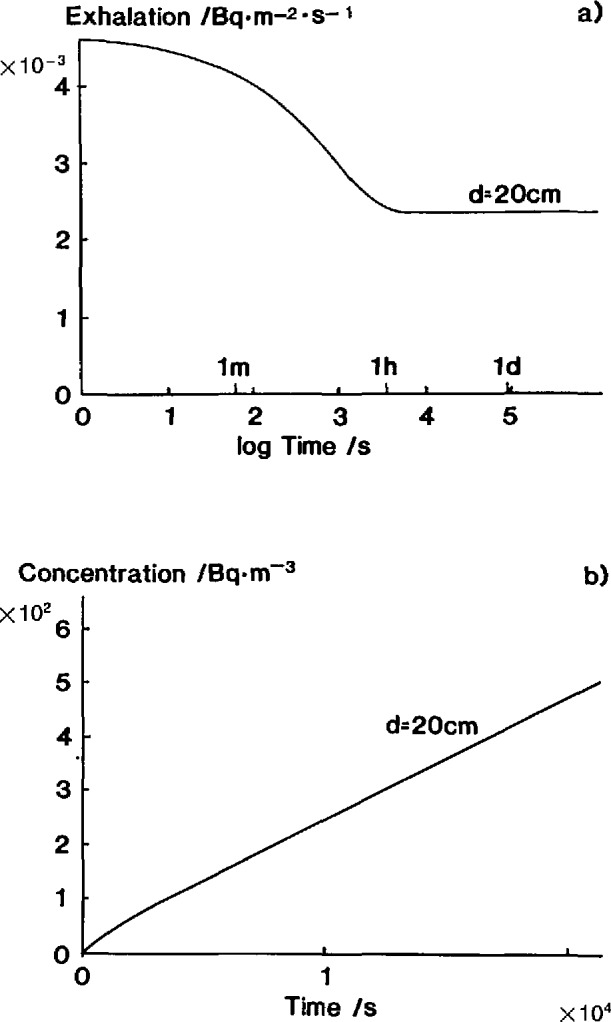
The temporal evolution of a) radon exhalation rate and b) the corresponding radon growth in the outer volume after closing a radon-tight accumulator. Diffusion length=2 m. (Theory).

A 20-cm thick sample in an accumulator of height 30 cm will typically exhibit the exhalation rate illustrated in figure 1 if the thickness, *d*, is less than the diffusion length, *L*. The corresponding cumulated concentration curve [fig. 1(b)] will, in an experimental situation, falsely give the impression of being linear.

**Figure 2:**
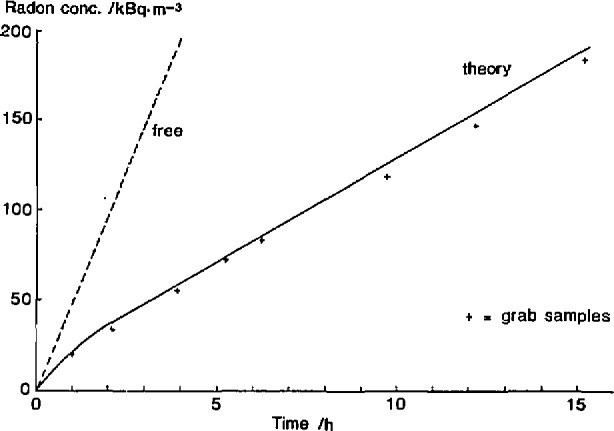
Radon concentration growth from enclosed dry sand mixed with 11% ground uranium ore by weight. Radon diffusion length =1.4 m, emanation fraction=0.33, radium−226 concentration = 1.2 kBq kg^−1^, and bulk density = 1710 kg m^−3^.

In order to reveal the rapid initial change in exhalation rate experimentally one has to follow the radon concentration build-up in the enclosed air volume more or less continuously. In figure 2 theoretical and experimental radon values for a 26-cm thick sample in an accumulator of height 30 cm are compared. The slope of the long linear part of the experimental curve in figure 2 corresponds to the final steady state exhalation rate, in this case about four times less than the free undisturbed exhalation rate.

Thin samples (*d* <0.5*L*) exhibit a final exhalation rate *E_∞_* that is related to the free exhalation rate *E*_f_ by the formula

$$\frac{E_{f}}{E_{\infty }}=1+\frac{1}{\alpha \gamma }$$ (2)

where

α = volume of the air above the sample divided by the pore volume of the sample,
*γ* = relative leakage factor [*γ*=(ν+λ)/λ],
*ν*=leakage rate constant of enclosure (s^−1^).

Choosing an outer air volume at least 10 times larger than the pore volume of the sample means that the depression of the free exhalation rate after closing the accumulator is less than 10%, a value acceptable in most experimental situations. If for reasons of sensitivity one has to work with small outer volumes, the initial drop in exhalation rate can be significantly reduced, but not completely avoided, if the outgassing radon is trapped in a charcoal trap or by other means in a flow-through system.

In cases where the change from free exhalation rate to final steady state exhalation rate is rapid enough (cf. fig. 1), eq (2) can be used to calculate the free exhalation rate because the slope of the radon growth curve is linear and corresponds to the steady state exhalation rate *E_∞_*.

## 3. Conclusions

In general it is not relevant to fit simple exponential formulas to results from closed-can exhalation measurements.

Certain sample/accumulator geometries are definitely unsuitable for free exhalation determination unless the radon growth is monitored more or less continuously and the results are interpreted by means of a time-dependent diffusion theory.

The mean exhalation rate during the first hours is approximately equal to the free and undisturbed exhalation only if the outer volume of air is much larger than the pore volume or the sample thickness is much larger than the radon diffusion length.
